# A Case Report of Catatonia and Neuroleptic Malignant Syndrome With Multiple Treatment Modalities: Short Communication and Literature Review

**DOI:** 10.1097/MD.0000000000001752

**Published:** 2015-10-30

**Authors:** Yu-Jie Chiou, Yu Lee, Chin-Chuen Lin, Tiao-Lai Huang

**Affiliations:** From the Department of Psychiatry, Kaohsiung Chang Gung Memorial Hospital and Chang Gung University College of Medicine, Kaohsiung, Taiwan.

## Abstract

We describe a case with complicated clinical presentations who was difficult to treat. We described the possible etiologies and differential diagnosis of neuroleptic malignant syndrome (NMS), catatonia, and infection, in details. This patient was also referred to neuro-intensive care unit for extensive workup and treatments by neurologist guidelines. In addition, we also used lorazepam–diazepam protocol and antipsychotics, but both failed to completely relieve her symptoms. She eventually responded to electroconvulsive therapy (ECT).

A 60-year-old female patient with schizophrenia was diagnosed to suspected pneumonia, urinary tract infection, and retarded catatonia at first. The brain computed tomography revealed no significant finding. She developed NMS caused by the administration of low-dose quetiapine (200 mg) after carbamazepine was discontinued. The Francis–Yacoub NMS rating scale (F-Y scale) total score was 90. We utilized lorazepam–diazepam protocol and prescribed bromocriptine and amantadine, but NMS was not improved. Meanwhile, we arranged the brain magnetic resonance imaging to survey the physical problem, which revealed agenesis of septum pellucidum and dilated lateral ventricles. She was then transferred to the neuro-intensive care unit on the 15th hospital day for complete study. The results of cerebrospinal fluid study and electroencephalography were unremarkable. She was transferred back to psychiatric ward on the 21st hospital day with residual catatonic and parkinsonian symptoms of NMS, and the F-Y scale total score was 63. Finally, her residual catatonic condition that followed NMS got improved after 11 sessions of ECT. On the 47th hospital day, the F-Y scale total score was 9.

This report underscores that the ECT is an effective treatment for a patient of NMS when other treatments have failed.

## INTRODUCTION

Neuroleptic malignant syndrome (NMS), first described by Delay et al in 1960^1^ related to exposure to haloperidol, is a potentially lethal adverse response to antipsychotic drugs, that is, characterized by altered consciousness, hyperpyrexia, muscular rigidity, and autonomic instability.^1^ Nowadays, NMS remains a significant source of morbidity and mortality among patients receiving antipsychotics, and often requires transfer to an intensive care unit, potentially interrupting psychiatric care.^2^ Because of increased awareness of NMS, earlier diagnosis of this drug-induced reaction, more conservative prescribing patterns, and the shift to use of atypical antipsychotics, the incidence of NMS was on the decline from 3% to 0.01%–0.02%.^3^ Sometimes, NMS is difficult for differential diagnosis with catatonia and infection. Here, we present the case of a patient with suspected quetiapine and carbamazepine related NMS, who recovered after electroconvulsive therapy (ECT), when other medical treatments had failed.

## CASE PRESENTATION

This 60-year-old single woman was a case of schizophrenia with initial presentation of persecutory delusion, auditory hallucination, and somatic delusion since age 28 and she lived with her sister. Despite regular follow-up and fair medical compliance, her psychotic symptoms exacerbated every 1 to 2 years, resulting in inpatient treatments. Over the past 30 years, her occupational and interpersonal functions deteriorated, while she could maintain fair personal care when her psychotic symptoms were under control. She was treated with quetiapine (925 mg/day) and carbamazepine (600 mg/day) after the last hospitalization in November, 2014. Over the next couple months, her quetiapine was tapered to 200 mg/day and carbamazepine was discontinued due to worsening depressed mood at the outpatient department.

In January 2015, she was observed to have mutism, stupor, negativism, muscle rigidity, poor intake, stereotypic grasping, staring, and dysuria for 3 weeks. She was sent to a local hospital and hospitalized for 8 days due to suspected aspiration pneumonia and urinary tract infection (UTI). She did not receive haloperidol intramuscular injection during hospitalization. She was prescribed quetiapine (200 mg/day) and carbamazepine (400 mg/day) in the local hospital. After antibiotic treatment of pneumonia and UTI, she was transferred to our acute psychiatric ward in a medical center due to persistence in mutism, stupor, negativism, muscle rigidity, poor intake, stereotypic grasping, and staring despite improvement of infection. Catatonia was suspected. Foley catheter and nasogastric tube were also needed since the hospitalization in the local hospital. She had no systemic disease. She had never used illicit substance before.

**FIGURE 1:**
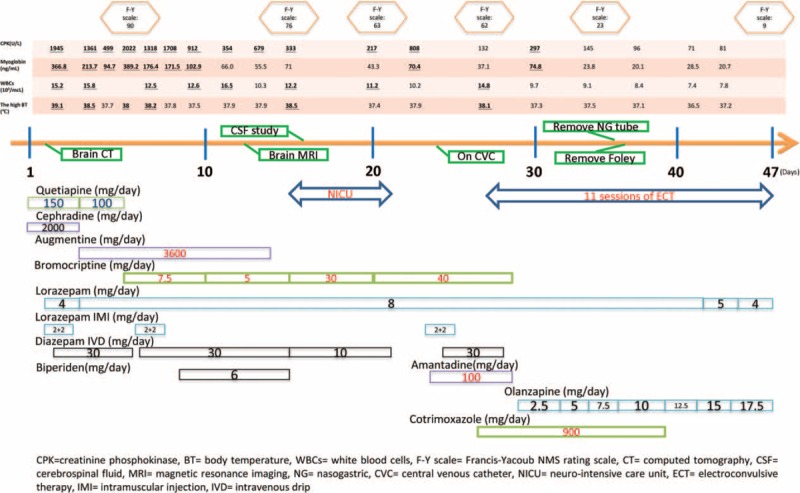
Medications record during this hospitalization. BT = body temperature; CPK = creatinine phosphokinase, CSF = cerebrospinal fluid, CT = computed tomography, CVC = central venous catheter, ECT = electroconvulsive therapy, F-Y scale = Francis-Yacoub NMS rating scale, IMI = intramuscular injection, IVD = intravenous drip, MRI = magnetic resonance imaging, NG = nasogastric, NICU = neuro-intensive care unit, WBCs = white blood cells.

After admission to our psychiatric ward, carbamazepine was discontinued and quetiapine was tapered down to 150 mg/day because of persistent catatonia (Figure 1). We started lorazepam at 4 mg/day orally for retarded catatonia. Her fever had subsided for 4 days before admission, and the chest X-ray showed no obvious cardiopulmonary disease. Antibiotic treatment for UTI was continued with cephradine 2000 mg/day orally. She developed hyperthermia (39.1 °C), diaphoresis, autonomic hyperactivity, and elevated levels of creatine phosphokinase (CPK = 1945 U/L), myoglobin (366.8 ng/mL), and white blood cells (15,200/mcL) on the 2nd hospital day. We checked free T4 and thyroid-stimulating hormone to rule out thyroid disorder, and they were within the normal ranges. Brain computed tomography revealed no acute intracranial pathology. Catatonia was impressed. Quetiapine was therefore tapered down to 100 mg/day. We then consulted the infectious disease specialist, who suggested changing antibiotic from cephradine to augmentin (a combination antibiotic containing 500 mg amoxycillin and 100 mg clavulanic acid) 3600 mg/day for upper respiratory tract infection. We checked the sputum acid-fast stain, 3 mycobacterial smears, sputum cultures, and mycobacterium tuberculosis polymerase chain reaction, and the results did not favor mycobacteria infection. We also titrated lorazepam up to 8 mg/day orally and utilized lorazepam–diazepam protocol, which consists of intramuscular injection of 4 mg of lorazepam during the first 2 hours followed by intravenous dripping of 10 mg diazepam in a 500 mL normal saline continuously to manage her NMS, a malignant form of catatonia. CPK and myoglobulin levels declined to 499 U/L and 94.7 ng/mL, respectively, on the 5th hospital day. However, fever (38 °C), muscle rigidity, diaphoresis, high blood pressure (190/98 mmHg), and tachycardia (118 beats/min) deteriorated on the 6th hospital day. Laboratory investigation revealed elevated CPK level 2022 U/L, myoglobin level of 389.2 ng/mL. The neurologist consultation confirmed our suspicion of NMS. The Francis–Yacoub NMS rating scale (F-Y scale) total score was 90. We immediately discontinued quetiapine and started bromocriptine at 7.5 mg/day for NMS. We had also initiated biperiden at 6 mg/day orally to treat suspected extrapyramidal symptoms on the 9th hospital day. She still had intermittent febrile episodes, severe rigidity, and lab data showed gradually decreasing CPK and myoglobin levels. The serum iron concentration was lower than the normal range (32.0 μg/dL) on the 11th hospital day, then increased to 62.0 μg/dL after administering intravenous iron sucrose complex for 3 days (total 350 mg iron). Brain magnetic resonance imaging was arranged to survey the physical problem, which revealed agenesis of septum pellucidum and dilated lateral ventricles. We discontinued augmentin after the remission of upper respiratory tract infection symptoms. Because of the lack of significant improvement of her NMS, she was transferred to the neuro-intensive care unit (Neuro ICU) to rule out central nervous system (CNS) infection on the 15th hospital day. The F-Y scale total score was 76.

In Neuro ICU, biperiden was discontinued, and bromocriptine was titrated up to 30 mg/day on the 15th hospital day then to 40 mg/day on the 20th hospital day. We checked the procalcitonin level on the 15th hospital day, and the low procalcitonin level (<0.09 ng/mL) indicated that sepsis was less likely. We tapered down intravenous diazepam from 30 to 10 mg/day and kept lorazepam 8 mg/day orally for catatonia. The results of cerebrospinal fluid study, including gram stain, acid fast stain, Indian ink stain, common aerobic/anaerobic culture, mycobacterium tuberculosis polymerase chain reaction, mycobacterium tuberculosis culture, virus, rapid plasma regain, herpes simplex virus DNA, herpes simplex virus IgG/IgM, cryptococcus, fungus culture, showed negative finding. Electroencephalography showed no abnormal slowing, no interictal epileptiform discharges. Then she was transferred back to psychiatric ward on the 21st hospital day. The F-Y scale total score was 63.

She was still observed to have mutism, stupor, negativism, muscle rigidity, poor intake, stereotypic grasping, staring, and dysuria, but hyperthermia and diaphoresis subsided. We inserted the central venous catheter in her right internal jugular vein due to difficulty setting the peripheral line on the 24th hospital day. We kept bromocriptine 40 mg/day, utilized lorazepam–diazepam protocol again, and prescribed amantadine 100 mg/day on the 23rd hospital day, but the residual catatonic state of NMS was not improved on the 27rd hospital day. The F-Y scale total score was 62 on the 27th hospital day. Fever (38.6 °C) and pyuria were noted on the 26th hospital day and we had prescribed co-trimoxazole (a combination antibiotic containing trimethoprim and sulfamethoxazole) at 900 mg/day to treat UTI for 2 weeks. Because of persistence of the residual catatonic state, ECT was initiated on the 27th hospital day. Anesthesia was induced with intravenous thiamylal 150 mg. The patient received 3 sessions of ECT per week. After 5 sessions of ECT, the symptoms of muscle rigidity, mutism, stereotypic grasping, and staring partially improved. Olanzapine was initiated at 2.5 mg/day for suspected psychotic symptoms on the 29th hospital day and steadily elevated to 17.5 mg/day for auditory hallucination, persecutory delusion. We subsequently removed nasogastric tube, Foley catheter, and discontinued lorazepam. UTI was improved. The F-Y scale total score was 23. After 11 sessions of ECT, the patient could communicate fluently, showing clearly oriented consicousness, and euthymic mood. No more stereotypical behaviors, muscle rigidity, psychotic symptoms, or unsteady gaits was detected. There were no adverse effects related to ECT, such as fracture, cardiopulmonary events, dental and tongue injuries, and memory impairment. The F-Y scale total score was 9 on the 47th hospital day. She was discharged a few days later with no symptoms of NMS. In the follow-up examination 1 month later, the NMS remained remitted.

## METHODS

Informed consent was obtained from the patient for the publication of this case report. The study was approved by the Chang Gung Memorial Hospital Institutional Review Board.

## DISCUSSION

Concerning the strategies we managed in this case, there were 3 steps taken to overcome the arduous clinical course. These steps included: ascertaining the diagnosis of NMS, complete study in Neuro ICU, and ECT after other treatment algorithm failure.

### Ascertaining the Diagnosis of NMS

Regarding the course of disease before admission, the possibility of catatonia caused by general medical condition (infection) that progressed to malignant catatonia, similar to NMS, seems reasonable because she had preceding catatonic signs (mutism, stupor, negativism, muscle rigidity, hands stereotypic grasping, and staring). These 2 disorders can be difficult to distinguish in the acute inpatient setting or in the intensive care unit.^4^ A review of 292 cases of malignant catatonia also indicated that the clinical features were indistinguishable from NMS in more than 20% of cases.^5^ On the other hand, differentiating catatonia from delirium is important, as catatonia is treated with benzodiazepines (BZDs) whereas delirium may be aggravated by BZDs. The lack of motor and speech abnormalities could help us to exclude delirium.^6^

There are many case reports of NMS associated with quetiapine.^7^ In most of the cases reported, the NMS is caused either after a coadministration of quetiapine with other medication or when other medical conditions have been involved.^8^ A possible mechanism could be that the hepatic enzyme cytochrome P450 3A4, which played a major role in the metabolism of quetiapine, could be induced, and the blood concentration of quetiapine decreased. Initially, the patient was prescribed of both quetiapine and carbamazepine. Carbamazepine could also reduce the plasma levels of many antipsychotics, including chlorpromazine, haloperidol, and clozapine.^9–12^ Her carbamazepine was discontinued during her infection, causing a rebound of quetiapine concentration despite continuous tapering, resulting in NMS. Furthermore, her infectious and catatonic conditions could be correlated with the incidence of NMS. These are our speculations but the true mechanism remains unknown.

Nowadays, BZDs are thought to be the first-line treatment for catatonia, which display a fast and dramatic improvement in symptoms,^13–15^ and demonstrate a response rate of 60% to 80% for catatonia.^16–19^ Previous studies indicated that low serum iron levels in patients with catatonia have been associated with treatment resistance to BZDs.^19,20^ NMS has been considered to be malignant form of catatonia, and may have a lower response rate to BZDs. In our case, which exhibited the low serum iron level, we repeatedly utilized the lorazepam–diazepam protocol for 3 times, which can rapidly relieve retarded catatonia in schizophrenia,^15^ but the response in this case was limited.

Bromocriptine has the dopaminergic effect. A review article demonstrated that the addition of bromocriptine to supportive measures excellently shortened the mean time to clinical response from 6.8 to 1.03 days.^21^ In addition, amantadine is a dopaminergic and anticholinergic agent, which could significantly reduce the NMS-related death rate according to a case–control analysis.^22^ In our case, we prescribed bromocriptine and amantadine but they also showed limited improvement on this patient's NMS. Our managements were in line with the previous treatment algorithm for NMS, including clinical presentation by illness stage or severity.^2^

### Complete Study in Neuro ICU

Differential diagnosis is pivotal because NMS is a diagnosis of exclusion. In addition to thyrotoxicosis and sepsis, which could be excluded via the laboratory studies, we should take CNS infections into consideration, especially viral encephalitis, which can be hard to distinguish from NMS. According to negative results of CSF studies, electroencephalography and neuroimaging, the possibility of CNS infection was low.

CPK level might stay within normal range if rigidity is not well developed, particularly in the early stage of the syndrome, or after the rigidity was improved by the BZDs treatment. Elevated CPK, especially within mild to moderate range, is a fairly nonspecific phenomenon and often observed in acutely psychotic patients due to intramuscular injections, physical restraints, intense isometric activity, and also for no apparent reason.^23,24^ In our case, the BZDs treatment, including the intravenous diazepam and intramuscular lorazepam, could relieve the severity of rigidity. Therefore, the elevation of CPK level did not correlate significantly with the clinical severity.

### ECT After Other Treatment Algorithm Failure

NMS is usually a self-limited disorder, with most episodes resolve within 2 weeks. Reported mean recovery times are 7 to 11 days.^25,26^ Catatonic and Parkinsonian symptoms of NMS may persist as a residual state lasting for weeks to months after more fulminant acute symptoms attenuate.^27,28^ In our case, ECT could shorten the course of the residual catatonic state that follows NMS and decrease the comorbidity, especially when pharmacological treatment with agents such as dopamine agonists, BZDs had failed.^29^

After recovery from an NMS episode, reintroducing antipsychotic treatment has been associated with a risk of NMS recurrence.^30,31^ However, most patients who need antipsychotic treatment can be safely treated under great precaution.^25^ A case report also demonstrated successful rechallenge of olanzapine for psychotic symptoms 5 days after NMS resolution,^19^ which was faster than in another report that put off its antipsychotic treatment until at least 2 weeks after recovery from NMS.^2^ In this case, we started olanzapine from relatively low doses (2.5 mg/day) with slow titration up to 17.5 mg/day over 18 days, and carefully monitored for early signs of NMS. If the treatment of olanzapine fails, we would consider clozapine, which causes less extrapyramidal symptoms.

## CONCLUSION

We described a case with complicated clinical presentations who was difficult to treat. We described the possible etiologies and differential diagnosis of NMS, catatonia, and infection, in details. We also used lorazepam–diazepam protocol and antipsychotics, but both failed to completely relieve her symptoms. She eventually responded to ECT.
